# Differential pain response at local and remote muscle sites following aerobic cycling exercise at mild and moderate intensity

**DOI:** 10.1186/s40064-016-1721-8

**Published:** 2016-01-28

**Authors:** Peter S. Micalos, Lars Arendt-Nielsen

**Affiliations:** School of Biomedical Sciences, Charles Sturt University, Panorama Ave, Bathurst, NSW 2795 Australia; Department for Health Sciences and Technology, Center for Sensory-Motor Interaction, School of Medicine, Aalborg University, Fredrik Bajers Vej 7-D3, Bld. D3, 9220 Ålborg, Denmark

**Keywords:** Hypoalgesia, Cycling exercise, Peak oxygen uptake, Pressure pain threshold

## Abstract

Physical exercise has been shown to inhibit experimental pain response in the post-exercise period. Modulation of the pain system may be differentiated between muscle sites engaging in contractile activity. The purpose of this study was to assess the pain response at remote and local muscle sites following aerobic exercise at different work intensities. Participants included 10 healthy and physically active males (mean age ± SD, 21.2 ± 3.4). Somatic pressure pain threshold (PPT) at the rectus femoris (local) and brachioradialis (remote) muscle site was measured at before (Pre), 5 min after (Post1), and 15 min after (Post2) aerobic cycling exercise at 70 and 30 % of peak oxygen uptake (VO_2peak_) performed on different occasions in a counterbalanced order, separated by minimum of 3 days interval. Repeated measures ANOVA for PPT reveals significant main effect for time (f = 3.581, *p* = 0.049, observed power = 0.588) and muscle site (f = 17.931, *p* = 0.002, observed power = 0.963). There was a significant interaction shown for exercise intensity by time (*f* = 11.390, *p* = 0.012, observed power = 0.790). PPT at rectus femoris following cycling exercise at 70 % of VO_2peak_ reveals a significant increase between Pre-Post1 (*p* = 0.040). PPT for rectus femoris following cycling exercise at 30 % of VO_2peak_ revealed a significant decrease between Pre-Post1 (*p* = 0.026) and Pre-Post2 (*p* = 0.008). The PPT for brachioradialis following cycling exercise at 30 % of VO_2peak_ revealed a significant decrease between Pre-Post1 (*p* = 0.011) and Pre-Post2 (*p* = 0.005). These results show that aerobic exercise increases PPT locally at the exercise muscle site following exercise at 70 % of VO_2peak_ but reduces PPT following exercise at 30 % of VO_2peak_.

## Background

Physical exercise has been shown to modulate experimental pain response in the post-exercise period (O’Connor and Cook 1999). Aerobic exercise such as cycling and running has been shown to reduce the sensitivity to experimental pain after exercise which returns to baseline within 30 min (Hoffman et al. 2004). The level of exercise to elicit pain inhibition in the post exercise period requires an intensity of 60–75 % of maximum oxygen consumption (Koltyn 2002) or at 50 % of heart rate (HR) reserve (Naugle et al. 2014). Hypoalgesic effects have been shown with exercise duration of 30 min but not at 10 min (Hoffman et al. 2004). Research on the effect of exercise on pain response is of interest due to evaluating changes in the function of the pain inhibitory system (Micalos et al. 2014). Understanding the function of the pain inhibitory system is beneficial for improving the prescription of exercise in chronic pain therapy (Carbonell-Baeza et al. 2010).

Experimental pain assessment requires the application of a controlled noxious stimulus to evaluate pain responses before and after intervention such as physical exercise. Experimental pain stimuli such as somatic mechanical pressure are applied in pain research settings to assess the function of the pain system (Kosek and Lundberg 2003). The pressure pain threshold (PPT) is the minimum required somatic stimulus intensity applied on the body surface to evoke a pain response (Fischer 1987; Cathcart and Pritchard 2006; Walton et al. 2011). Assessment of PPT involves application of an increasing pressure stimulus on the skin by an algometer until the signal for pain is conveyed (Ohrbach and Gale 1989). Previous research indicates that aerobic exercise reduces pain sensitivity (Hoffman et al. 2004) and increases the PPT after exercise (Koltyn et al. 1996) in healthy participants.

The mechanism underlying the exercise-induced pain inhibition has not been fully elucidated (Koltyn et al. 2014). It has been shown that the hypoalgesia following exercise is evident throughout the body and regionally at the exercise muscle site (Kosek and Lundberg 2003). This suggests that physical exercise activates central descending pathways for widespread pain inhibition, however, this effect is also augmented by localised pain inhibitory mechanisms at the exercising muscle site. In support of this, it has been shown that aerobic exercise increases the PPT to a greater extent at the site of the exercising body part (Vaegter et al. 2014). However, the effect of different exercise intensities has not been fully elucidated together with regional pain inhibition. Therefore, the purpose of this study is to assess the pain threshold at local and remote muscle sites following aerobic exercise at different intensities to investigate the balance between regional and widespread somatic pain sensitivity.

## Results

Mean age, height, body weight, and VO_2peak_ (±SD) of participants are shown in Table 1. Mean power output (±SD) for submaximal exercise at 70 and 30 % of VO_2peak_ was 186 ± 24 and 84.5 ± 15.4 W, respectively. The mean time interval (±SD) between the sessions was 6.3 ± 3.3 days. The mean HR and RPE response at 70 % VO_2peak_ was 151.8 ± 16.7 beats/min and 13.5 ± 1.3 units, respectively. At 30 % VO_2peak_ the mean HR and RPE was 106.4 ± 20.4 beats/min and RPE 9.9 ± 3.2 units.

**Table 1:** Participant characteristics

Age (years)	Height (cm)	Mass (kg)	VO_2peak_ (l/min)
21.2 (±3.4)	179.5 (±5.8)	77.1 (±9.3)	3.5 (±0.4)

Results for Pre-exercise intra-class correlation coefficient show 0.63 for brachioradialis and 0.67 for rectus femoris muscles, respectively. Repeated measures ANOVA for PPT reveals significant main effect for time (f = 3.581, *p* = 0.049, observed power = 0.588) and muscle site (f = 17.931, *p* = 0.002, observed power = 0.963). There was a significant interaction shown for exercise intensity by time (*f* = 11.390, *p* = 0.012, observed power = 0.790).

**Fig. 1:**
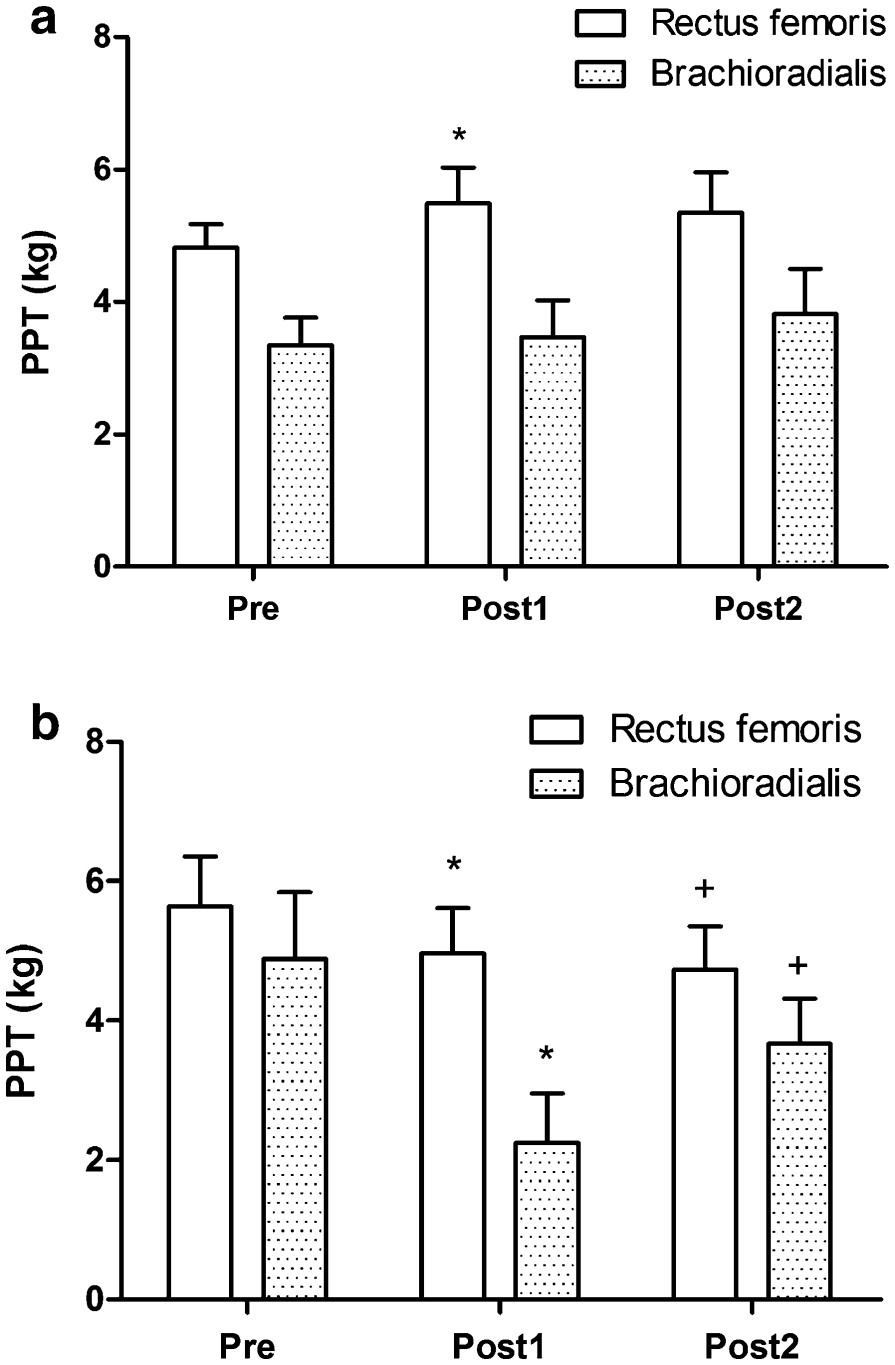
Changes in pressure pain threshold at the rectus femoris (local) and brachioradialis (remote) muscle site following aerobic cycling exercise at 70 % (**a**) and 30 % (**b**) of VO_2peak_. *p < 0.05, Pre-Post1; ^+^ *p* < 0.05, Pre-Post2

Based on results for main effects, separate analyses were performed for exercise intensity. The mean PPT at the rectus femoris and brachioradialis sites following aerobic exercise at 70 % of VO_2peak_ are shown in Fig. 1a. PPT at rectus femoris following cycling exercise at 70 % of VO_2peak_ reveals a significant increase between Pre-Post1 (*p* = 0.040) but not for Pre-Post2 (*p* = 0.131). The PPT for brachioradialis following cycling exercise at 70 % of VO_2peak_ reveals no significant difference between Pre-Post1 and Pre-Post2.

The PPT following aerobic exercise at 30 % of VO_2peak_ at the rectus femoris and brachioradialis muscle sites are shown in Fig. 1b. PPT for rectus femoris following cycling exercise at 30 % of VO_2peak_ reveals a significant decrease between Pre-Post1 (*p* = 0.026) and Pre-Post2 (*p* = 0.008). The PPT for brachioradialis following cycling exercise at 30 % of VO_2peak_ reveals a significant decrease between Pre-Post1 (*p* = 0.011) and Pre-Post2 (*p* = 0.005).

## Discussion

The present results reveal an increase in PPT at the local exercise muscle site following cycling exercise at 70 % of peak oxygen uptake. Our results comply with previous research showing that aerobic exercise induces hypoalgesic effects at the exercising muscle site compared to the non-exercise site (Vaegter et al. 2014). These results are also in accord with previous research showing increased PPT following aerobic exercise at high intensity compared to low intensity exercise (Vaegter et al. 2014). However, the present results did not show a significant increase in PPT in the non-exercising muscle site following aerobic exercise at 70 % of peak oxygen uptake. This level of exercise intensity was selected in the present study to enable participants to complete 30 min of exercise and show experimental pain inhibition (Koltyn 2002; Hoffman et al. 2004). A recent study showed moderate exercise intensity was insufficient to increase the PPT at the non-exercise site but was adequate following vigorous intensity exercise (Naugle et al. 2014). Hence, the aerobic exercise at 70 % of peak oxygen uptake in the present study was below a vigorous intensity to induce widespread pain inhibition. Additionally, the increase in PPT was observed immediately after exercise but was not significant at 15 min post-exercise. Together, this suggests moderate exercise intensity induces a transient localised pain inhibition at the exercising muscle site but not for widespread pain inhibition at the non-exercising muscle site.

Potential mechanisms for increased pain threshold at the exercising muscle site are associated with peripheral inhibition of the nociceptive signal. Endogenous opioid-related substances are expressed at central sites in the nervous system but are also locally expressed at the exercising muscle site and this may attenuate the nociceptive signal (Tegeder et al. 2003). However, caution must be applied when appraising exercise-induced pain inhibition with experimental stimuli. It has been shown that noxious thermal sensitivity such as cold and heat are not consistently inhibited following aerobic exercise (Ruble et al. 2005; Janal et al. 1984). In another study, reduced pressure pain sensitivity was observed following isometric exercise but not for aerobic exercise (Vaegter et al. 2015). Moreover, heat pain threshold was shown to be increased but not PPT following aerobic exercise (Kodesh and Weissman-Fogel 2014). Reduced pain sensitivity following exercise has been more consistently evident with suprathreshold noxious stimuli (Kodesh and Weissman-Fogel 2014; Naugle et al. 2012). Together, this suggests that the reduced pain sensitivity following exercise may be observed according to the type of noxious stimulus and the modality of exercise applied (Naugle et al. 2012; Lau et al. 2015). Despite these limitations, our results show an increase in the PPT at the exercising muscle site following moderate intensity aerobic exercise.

The present results also revealed a decrease in PPT following mild exercise at 30 % of VO_2peak_. This mild exercise intensity was selected to enable a substantial difference in exercise responses between the moderate and mild intensity. The reduced PPT following exercise was unexpected, however, this has previously been demonstrated. In research by Lau et al. (2015), this shows a reduced PPT immediately after eccentric exercise. In contrast, no difference in pressure pain sensitivity following low intensity aerobic exercise at 50 % of maximum oxygen uptake has been reported (Vaegter et al. 2014; Hoffman et al. 2004). Reason for this disparity might be associated with the reduced exercise intensity in the present study which was set at 30 % of VO_2peak_. It is possible that mild exercise intensity may facilitate the somatosensory system and increase the sensitivity to noxious stimuli. Primary excitatory and inhibitory neurotransmitters in the central nervous system include glutamate and GABA, respectively. Several neurotransmitter substances have been shown to be centrally (Meeusen and De Meirleir 1995) and peripherally (Karlsson et al. 2015) released during exercise which can facilitate or inhibit pain signalling. In the animal model, enhanced GABA has been observed in the forebrain with regular exercise (Hill et al. 2008). The midbrain periaqueductal grey (PAG) and the rostral ventromedial medulla (RVM) have been shown to exert bidirectional facilitation and inhibition over nociceptive dorsal horn transmission (Fields et al. 2006). Using this paradigm, a threshold exercise stress potentially modulates central pain transmission by activating the PAG–RVM network (Micalos 2014). Activation of this network is associated with the central endogenous release of neurotransmitters including opioids, serotonin, and cannabinoids (Fields et al. 2006). However, it is possible that mild aerobic exercise may facilitate ascending afferent signalling compared to moderate intensity exercise which activates the descending pain inhibitory system. Additional research would be required to further elucidate these changes in pain sensitivity following exercise at different intensities.

Several limitations in this study need to be acknowledged. Firstly the present study did not include a separate non-exercise control group. Additionally, the intra-class correlation analysis between trials at baseline showed moderate levels of reliability compared to previous research showing moderate to excellent reliability for PPT (Walton et al. 2011). This previous research used three repeated assessments of PPT at each site under resting conditions whereas the present study included a single assessment at bilateral muscle sites. However, due to differences between trials we analysed data separately for each exercise intensity. The data extracted for the present results were part of a larger data set involving noxious stimuli, however, the counterbalanced testing would have offset any cross-over effects. It should be noted that the transient pain inhibition limits the capacity to perform several repeated pain threshold assessments following exercise. Moreover, the participant sample may not generalise to older participants, females, and chronic disease patients.

## Conclusion

In conclusion, these results show that aerobic activity attenuates pressure pain sensitivity locally at the exercise muscle site following cycling exercise at 70 % of peak oxygen uptake, however, may facilitate pain sensitivity following exercise at 30 % ofVO_2peak_. This indicates that localised pressure pain inhibition is shown at the exercise muscle site following moderate exercise but not for widespread pain inhibition.

## Methods

The study was conducted with the approval of the Charles Sturt University Ethics in Human Research Committee (Approval Number: 2007/107) and all participants signed a letter of informed consent. The data extracted in the present study is a separate part of a larger data set that has been previously published (Micalos et al. 2015).

### Participants

**Fig. 2:**
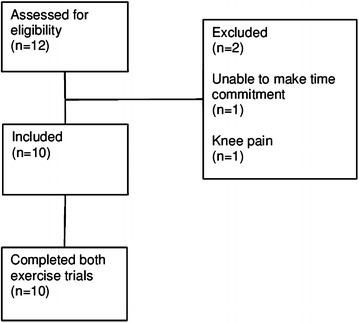
Flowchart of participant recruitment. Ten participants completed both exercise trials at 70 and 30 % of VO_2peak_ on separate occasions

Participants were recruited from a university student population (Fig. 2). Participants included ten healthy and physically active males. Exclusion criteria included the recent use of anti-inflammatory and analgesic medications, lower limb injury or surgery, acute infection, and chronic disease.

### Experimental design

We used a mixed design repeated measures. Each participant attended the laboratory on three separate visitations. The first visitation was required to measure anthropometric data and assess peak oxygen uptake (VO_2peak_). A second and third visitation was required to assess PPT before and after cycling exercise intensity at 70 and 30 % of VO_2peak_, on separate visitations. A minimum of 3 days interval between trials was conducted to avert delayed exercise muscle soreness. The succession of exercise intensities was counterbalanced to avert an order effect. Participants were instructed to maintain their regular daily activities and to avoid exercise on the day when the experimental sessions were performed.

### Assessment of peak oxygen consumption

The assessment of peak oxygen consumption was performed by ramped cycle exercise test (Jones et al. 2014). Peak exercise oxygen uptake was assessed by respiratory gas analyser (Par Medics, Pneumotach TrueOne 2400, Sandy, UT, USA) connected to a Hans Rudolph valve (Hans Rudolph Inc., Shawnee, KS, USA) during exercise on a stationary cycle ergometer (Lode, Sport Excalibur, Groningen, The Netherlands). The VO_2peak_ assessment began at 60 W of power output and was incremented by 30 W at 3 min intervals until exercise tolerance. The HR was assessed by telemetry (Polar RS200, Polar Electro, Kempele, Finland).

### Assessment of pressure pain threshold

The PPT was assessed before and after cycling exercise performed at 70 and 30 % of VO_2peak_ on two separate visitations in a counterbalanced order. PPT was assessed using a handheld pressure algometer (Force One, Wagner, USA) with a surface contact area of 1 cm^2^. The PPT was assessed by increasing the surface contact pressure until the participant verbally indicated that the stimulus was painful. All PPT assessments were performed by one experimenter over the muscle belly at the left and right rectus femoris (local) and brachioradialis (remote) muscle sites. Each participant was familiarised with the procedure prior to assessment of the PPT.

The PPT was assessed at before exercise (Pre), 5 min after exercise (Post1), and 15 min after exercise (Post2). Participants were seated in a reclined liftback table for the PPT assessments. Each aerobic exercise session was performed in a climate controlled laboratory at the same time of day to minimise diurnal variation. The duration of aerobic exercise was for 30 min on a bicycle (Avanti Bikes, Auckland, New Zealand) mounted on a stationary cycle trainer (Tacx Cycleforce Basic, Rotterdam, The Netherlands) with a power output display (SRM, Colorado Springs, USA). The exercise load required to maintain the designated power output was pre-determined at 70 or 30 % of VO_2peak_ as shown by the power output display unit. The HR and rating of perceived exertion (RPE) (Borg 1970) were assessed every 2 min during exercise.

### Statistical analysis

Intra-class correlation coefficient was used to determine PPT reliability for Pre-exercise at 70 and 30 % of VO_2peak_. Three-way repeated measures ANOVA with observed power analysis on PPTs were performed for exercise intensity (70 % VO_2peak_; 30 % VO_2peak_), muscle site (rectus femoris; brachioradialis) and time (Pre-Post1; Pre-Post2). Checks for data sphericity were performed and Greenhouse–Geisser procedure integrated where required. Where significant main effects were shown, paired samples statistics were performed on PPT’s at 70 and 30 % VO_2peak_ between Pre-Post1 and Pre-Post2. Data were analysed using IBM SPSS statistics v20. Results for PPT are presented as mean with standard error. Level of significance was set at *p* < 0.05.

## Abbreviations

HR: heart rate
PPT: pressure pain threshold
RPE: rating of perceived exertion
VO_2peak_: peak oxygen uptake
W: Watt
